# Pharmaceutical Company’s Choices of Indication for the First Clinical Projects in Oncological Drug Development in the United States

**DOI:** 10.1007/s43441-024-00718-2

**Published:** 2024-10-31

**Authors:** Can Wu, Shunsuke Ono

**Affiliations:** Laboratory of Pharmaceutical Regulatory Science, Graduate School of Pharmaceutical Sciences, The University of Tokyo, 7-3-1 Hongo, Bunkyo-ku, Tokyo 113-0033 Japan

**Keywords:** Oncological drug development, First developed indication, Lead indication, Cancer type, Company behavior

## Abstract

We analyzed factors shaping the choice of the lead indication (i.e., cancer type) in the first clinical development projects of new oncological drugs in the United States (US), and how the type of pharmaceutical company is related to this choice. We selected 576 new clinical development projects in the US since 2000 for analysis. These projects were characterized according to three potential perspectives detected by multiple correspondence analysis: the morbidity of the cancer type which corresponds to market size of each cancer type, the company’s previous experience with the cancer type, and the company’s attitude to development risks. Mega firms tend to choose cancer types with higher morbidity (and large-market), previously experienced cancer types, while diverse small firms choose both major and rare cancers and both high- and low-risk projects, indicating that different sizes of firms utilize different development entry patterns. Common tendencies concerning the choice of lead indication were found across all companies. Cancer types the company had developed and launched in the past were more likely to be chosen; cancer types with high five-year survival rates and those with high competition were less likely to be chosen. The study showed that pharmaceutical companies seem to enter clinical development from cancer types where they can demonstrate their strengths and advantages through experience, depending on each cancer type’s different market sizes and development difficulties. The results could provide clues for considering what support measures and incentives are appropriate to balance the efficiency of industrial development and the fulfillment of society’s unmet medical needs.

**Supplementary Information:**

The online version contains supplementary material available at 10.1007/s43441-024-00718-2.

## Introduction

Oncological drug development is one of the most important area in drug development. Cancer incidence is expected to rise significantly through 2050, and more than 2,000 new oncology clinical trials started in 2023 with novel modalities and significant promise for cancer treatment, including cell and gene therapies, antibody-drug conjugates, multi-specific antibodies, and radioligand therapies [1]. Many cancer types share common molecular pathways for tumorgenesis, cell proliferaion and metastasis [2]. So that, in most cases, a drug candidate is developed as potentially effective against multiple cancer types. Given the constraints of industrial research and development (R&D) resources and the different probability of development success for each cancer type, the order and timing of obtaining approval for each indication is an important component of lifecycle management (LCM) for pharmaceutical companies [3].

It is a crucial R&D decision for pharmaceutical companies to target and initiate the clinical development of a new oncological drug, given the science level pertinent to the product, medical needs, regulation, and economic and business prospects. The choice of lead indication is critical for a company’s development strategy. The market for anticancer drugs is fragmented by indication, and competition with rival products occurs in such fragmented markets. New molecular entities are granted marketing exclusivity in the US for five or seven years for the first indication approved, depending on orphan designation status. Companies can also benefit from the remaining effective patent period. From the perspective of obtaining regulatory approval, a common strategy is first to develop orphan indications, which are treated favorably in various ways upon approval, even though the market is small, and then expand the indications to other types of cancer with larger markets. The choice of the first indication thus plays a critical role in maximizing the probability of development success and promoting market penetration of the drug [4, 5].

The potential target indication of a new drug is determined by several factors that define the ‘druggability,’ including the physical and chemical properties of the drug and its pharmacological targetability considering the cancer biology. However, since it is the company that decides first the nature of the drug to be chosen for development, when chosing lead indication from several targetable cancer types which share common molecular pathways, the company’s decision to embark on the development project of the combination of a drug and an indication is based on business-related considerations supported by factors such as previously observed success rates in the same therapeutic fields, development costs, and expected benefits. The decision is also affected by the company’s strategic priority of the drug and/or indication.

Previous studies have discussed companies’ approaches to the development targets of anticancer drugs. Companies tend to choose lead indication that could be applied to specific regulatory schemes such as conditional marketing approval, expedited review, and orphan designation [3, 6–9]. These regulatory schemes generally target serious illnesses with high unmet clinical needs, allowing faster market access. Some studies focused on the strategies for extending indications, including those for LCM after marketing, which are usually related to the pricing strategy under the insurance system [5, 10].

Prior research on which cancer type to target as the lead indication has often examined the first approved indication (FAI) and not the first developed indication (FDI) [3, 5–10]. FAI is an important LCM indicator, but it is not necessarily an appropriate indicator of a company’s decision at a very early stage of clinical development, especially in oncology, where high failure rates are the norm [11, 12]. FAI reflects the consequence of various events that a company cannot control on its own and also decisions on the side of regulators. Analysis using FDI rather than FAI is necessary to prospectively explore the intentions and strategies of firms in the early development stages.

This research aimed to clarify FDI’s decision in the early development phase for recent oncology drugs in the US and describe factors (i.e., attributes of company and drug, market backgrounds) influencing the decision. Various companies populate the new drug development market in the US, and the mode of action of new drugs is also diversifying. We took such diversity in the development market as a cue to highlight the dynamics behind firms’ choice of lead indication. For this purpose, we first classified all first projects visually using multiple correspondence analysis (MCA) and explored how the firm type is related to the projects undertaken. We then used regression analysis to examine which firm and drug attributes and developmental backgrounds each FDI (i.e., cancer type) choice is likely to occur.

Clarifying the reality of decision on FDI, for which few previous studies exist, provides valuable clues not only from the perspective of corporate R&D strategy theories but also for considering the health consequences of the decision, that is, its impact on public health.

## Methods

We collected 1,592 oncological products from the database of Pharmaprojects (Citeline, https://citeline.informa.com/#/drugs; search date as of 3rd February 2022) for new molecular entities (NME), the development of which was conducted in the US. The search criteria were disease group (anticancer), drug country (USA), status reached (clinical phase). Products with unidentified pharmacological activity and products with no change on Pharmaprojects since year 2000 were excluded. We defined FDI as the first certain indication developed by a company. We excluded 225 products for which development was initiated before 2000 because searching for clinical trial information was not feasible. Clinical trial information was collected from the ClinicalTrials.gov (https://clinicaltrials.gov/), the European Union Clinical Trials Register (https://www.clinicaltrialsregister.eu/), the Japan Registry of Clinical Trials (https://jrct.niph.go.jp/), and the Chinese Clinical Trial Registry (https://www.chictr.org.cn/). All these clinical trials were ranged in time series and target indications were extracted per clinical trial.

**Fig. 1:**
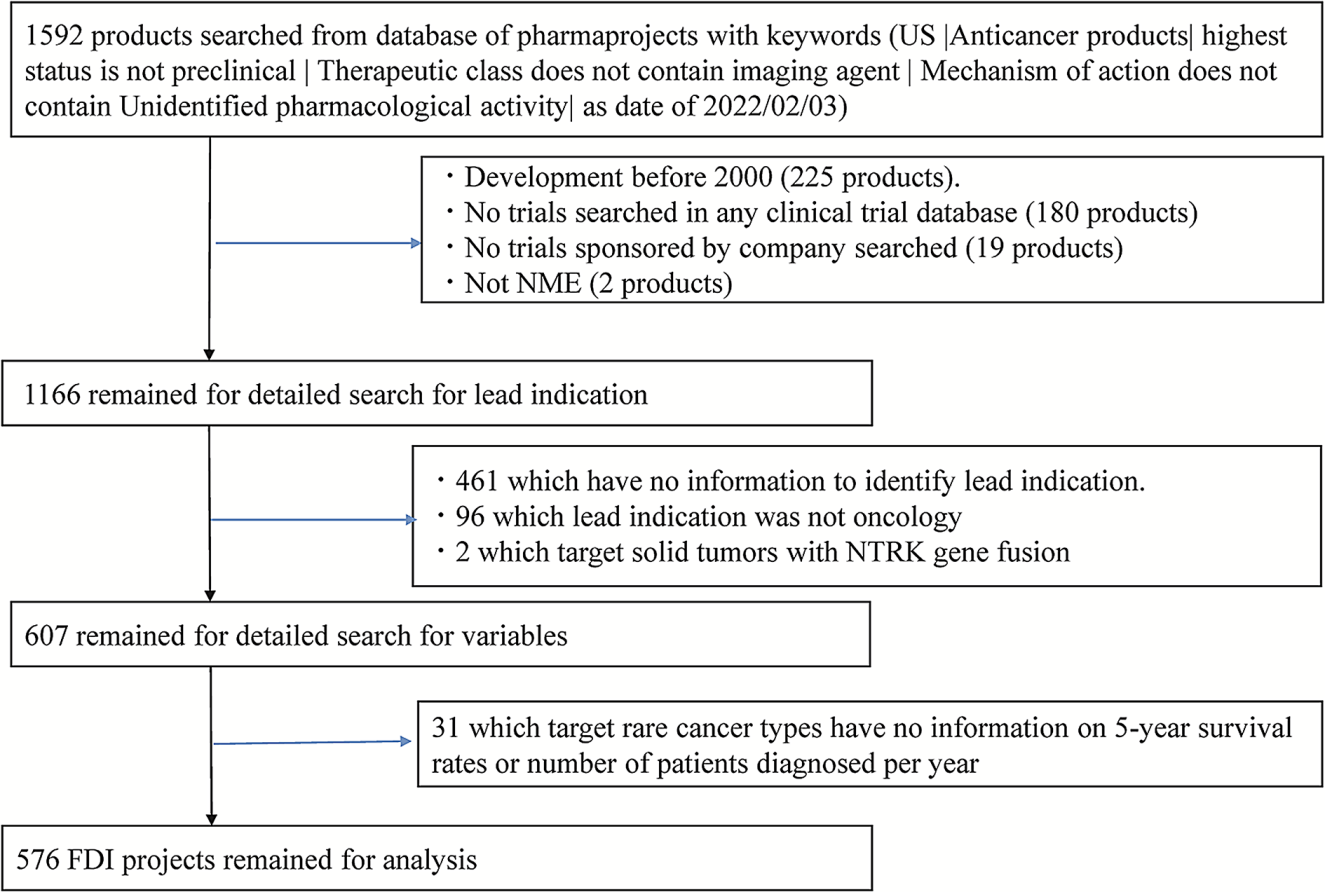
Flowchart of data collection

There were 180 products for which no clinical trials were registered in any of the above clinical trial databases. Clinical trials of 19 products were being conducted by academia only, and two products were examined as non-NME, so these products were excluded. Therefore, 1,166 products were extracted for further review of clinical trial information. For clinical trials sponsored by a pharmaceutical company, the first development projects and indications were identified for each product. FDI was defined as the first single indication a company conducted clinical trials. For Phase 1 dose-escalation trials targeting multiple indications for solid or blood cancers, FDI in this study was defined to be the single indication developed in the expansion part or the first Phase 2 trial phase. Among 1,166 products, 461 products were still in the Phase 1 dose-escalation stage for general solid or hematological cancers as of our search date. These products were excluded because FDIs were not identified. Also, 96 products for which FDI was not cancer and two products targeting neurotrophic tyrosine receptor kinase (NTRK) gene fusion mutated solid tumors rather than a single indication (entrectinib, larotrectinib) were excluded, resulting in a total of 607 products (projects) suited to our purpose of FDI exploration. However, for 31 projects, information on morbidity (number of patients diagnosed per year) or five-year survival rates relating to cancer types was unavailable. Our dataset for analysis contained 576 projects (Fig. 1). Morbidity is considered to have direct correlation with market size and can be the substitute variable of market size for each cancer type.

We collected information on individual firms, including global sales of a company at the time of development from compnanies’ annual report, annual filing of finalcial statements (i.e., forms10-K or 20-F) from the database of US Securities and Exchange Commission, the company’s experience in the approval in the same indication from Food and Drug Administration (FDA) approved drugs list, and previous experience in development in the same indication from the ClinicalTrials.gov, based on the available literature [13–16]. Drug characteristics were searched from the database of Pharmaprojects, e.g., mode of action (MOA), classification of target molecules, and drug modality chemical vs. biological. Five-year survival rates and number of patients diagnosed per year related to each cancer type were searched from Cancer.Net US (https://www.cancer.org/) and data of year 2021 was used. Observed success rates was used as general success rates observed definited as percentage of launced ones in overall products in the same therapeutic fields. Number of competing trials at the time of development) were collected from the ClinicalTrials.gov. Detailed data collecting methods and definication were described in Table S1.

We visualized the variability of projects in our sample using MCA. MCA enables the extraction of important dimensions that maximize the ‘variability’ of project features. We analyzed the display of category centroids on the detected axes and the plotting and display of the individual projects.

To explore the relationship between the cancer types chosen for the project and background factors, we performed regression analyses based on the conditional logit choice model (McFadden model). Two analysis models were applied to address two different assumptions. In the first model, we assumed companies could choose any cancer type; all cancer types observed in our dataset (25 cancer types) were set as possible. In the second model, we put a more practical assumption that companies could choose only one of the cancer types observed in the same MOA. Drugs belonging to an MOA for which only a single cancer type was chosen were excluded (Table S2).

A company’s development experience and competition were measured by the number of trials at the time of development, so the variables were different for each project. The number of patients diagnosed per year, observed success rates and five-year survival rates were set as variables specific to cancer type; therefore, these variables are the same across projects for the same cancer type. Non-small cell lung cancer (NSCLC) was set as the base indication because it is the most common FDI (see Table 1).

**Table 1:** Summary of first developed indiation (FDI) projects categorized by cancer type and company type

Cancer type	Number of projects (%)	Projects by mega firms	Projects by large firms	Projects by medium firms	Projects by small firms
Non-small-cell lung cancer (NSCLC)	84(14.6%)	23	7	3	51
Breast cancer	75(13.0%)	27	4	4	40
Acute myeloid leukemia (AML)	65(11.3%)	18	9	1	37
Prostate cancer	49 (8.5%)	12	1	1	35
Multiple myeloma (MM)	39 (6.8%)	11	4	3	21
glioblastoma	32 (5.6%)	10	0	0	22
Chronic lymphocytic leukemia (CLL)	28 (4.9%)	3	5	2	18
melanoma	28 (4.9%)	10	3	1	14
Colorectal cancer	19 (3.3%)	3	3	3	10
Pancreatic cancer	18 (3.1%)	2	0	0	16
Non-Hodgkin lymphoma	17 (3.0%)	6	1	0	10
Ovarian cancer	17 (3.0%)	4	1	0	12
Myelofibrosis (MF)	14 (2.4%)	4	0	0	10
Hepatocellular carcinoma	14 (2.4%)	1	2	3	8
Myelodysplastic syndromes (MDS)	13 (2.3%)	0	1	2	10
Small-cell lung cancer (SCLC)	13 (2.3%)	9	0	0	4
Renal cell carcinoma	9 (1.6%)	1	1	0	7
Bladder cancer	8 (1.4%)	1	0	1	6
Head and neck cancer	8 (1.4%)	1	0	1	6
Acute lymphocytic leukemia (ALL)	7 (1.2%)	3	0	0	4
Gastric cancer	6 (1.0%)	2	0	2	2
Chronic myeloid leukemia (CML)	4 (0.7%)	3	0	0	1
mesothelioma	4 (0.7%)	0	0	0	4
Endometrial cancer	4 (0.7%)	2	0	0	2
Cervical cancer	1 (0.2%)	0	0	0	1
Total	576	156	42	27	351

We conducted several analyses applying different sets of variables. Model 1–1 was the base model that did not include features specific to cancer types. Models 1–2, 1–3, and 1–4 included features specific to cancer types (Model 1–2 included morbidity, model 1–3 indluced observed success rates in the same indication and model 1–4 included 5-year survival rates). The number of competing trials, approval experience, development experience in the same indication, and cancer features were set as alternative-specific variables. In contrast, global sales of the company and drug features were set as case-specific variables in the analysis.

Stata version 17 was used for statistical analysis of MCA and regression analysis.

## Results

### Characteristics of First Clinical Development Projects

Overall, there were 576 first development projects that met our selection criteria for all the company sizes. 351 (60.9%) were small firms’ projects, and 156 (27.1%) were mega firms’ projects. Chemical entities were involved in 546 (94.8%) projects, and 30 (5.2%) projects were for biologics. There were 164 (28.5%) projects for new MOA drugs and 412 (71.5%) projects for follow-on drugs. Of the 83 (14.4%) products in our data set that the FDA approved, 32 (38.6%) had FAI that differed from FDI.

The indications for the 576 first development projects are shown in Table 1. NSCLC was chosen most often as FDI, accounting for 14.6% of the total. The highest three FDIs ranked were acute myeloid leukemia (AML) (11.3%), multiple myeloma (MM) (6.8%), and chronic lymphocytic leukemia (CLL) (4.9%) in hematological cancers and NSCLC (14.6%), breast cancer (13.0%), and glioblastoma (5.6%) in solid cancers. Most of the mega firm projects were implemented by US and European companies (50.6% and 45.5%, respectively, Table S3). More than half of the projects by large and medium firms were by Asian firms (52.4% and 51.9%, respectively). Of the small firm projects, 67% were undertaken by US companies.

**Fig. 2:**
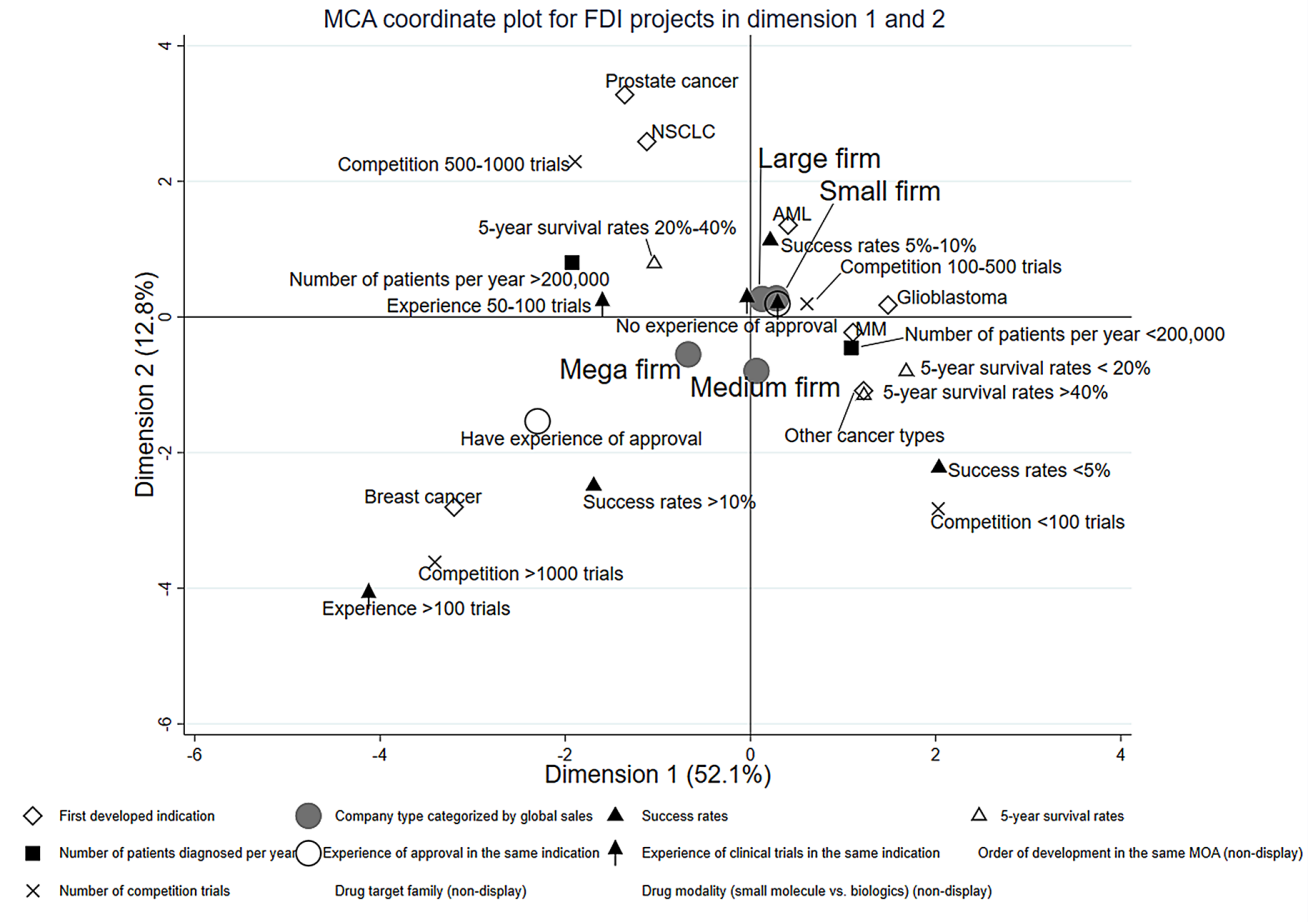
Multiple correspondence analysis coordinate plot for first developed indication (FDI) projects in dimension 1and 2

**Fig. 3:**
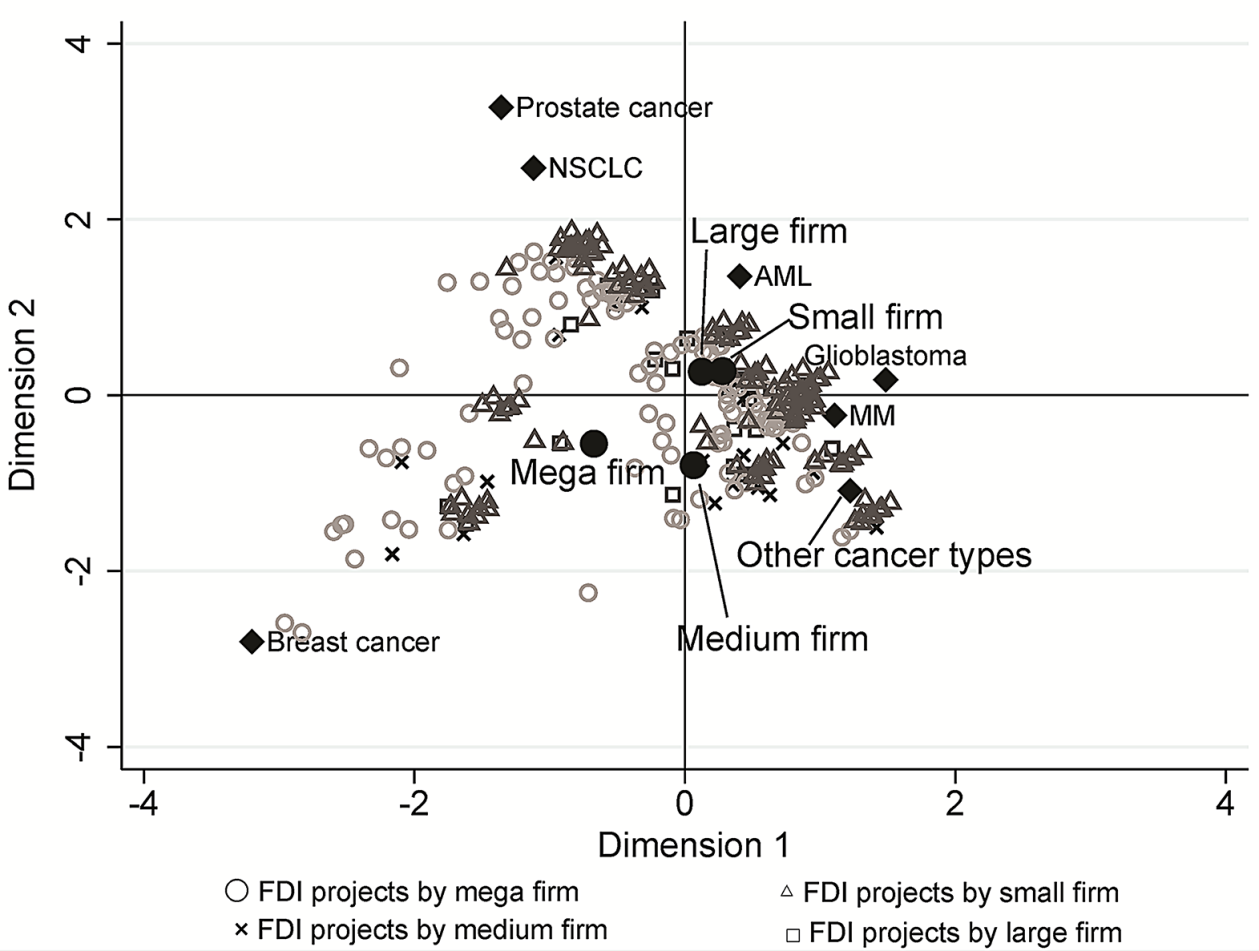
Plots of individual first developed indication (FDI) projects, cancer types and company types in multiple correspondence analysis dimension 1 and 2

**Fig. 4:**
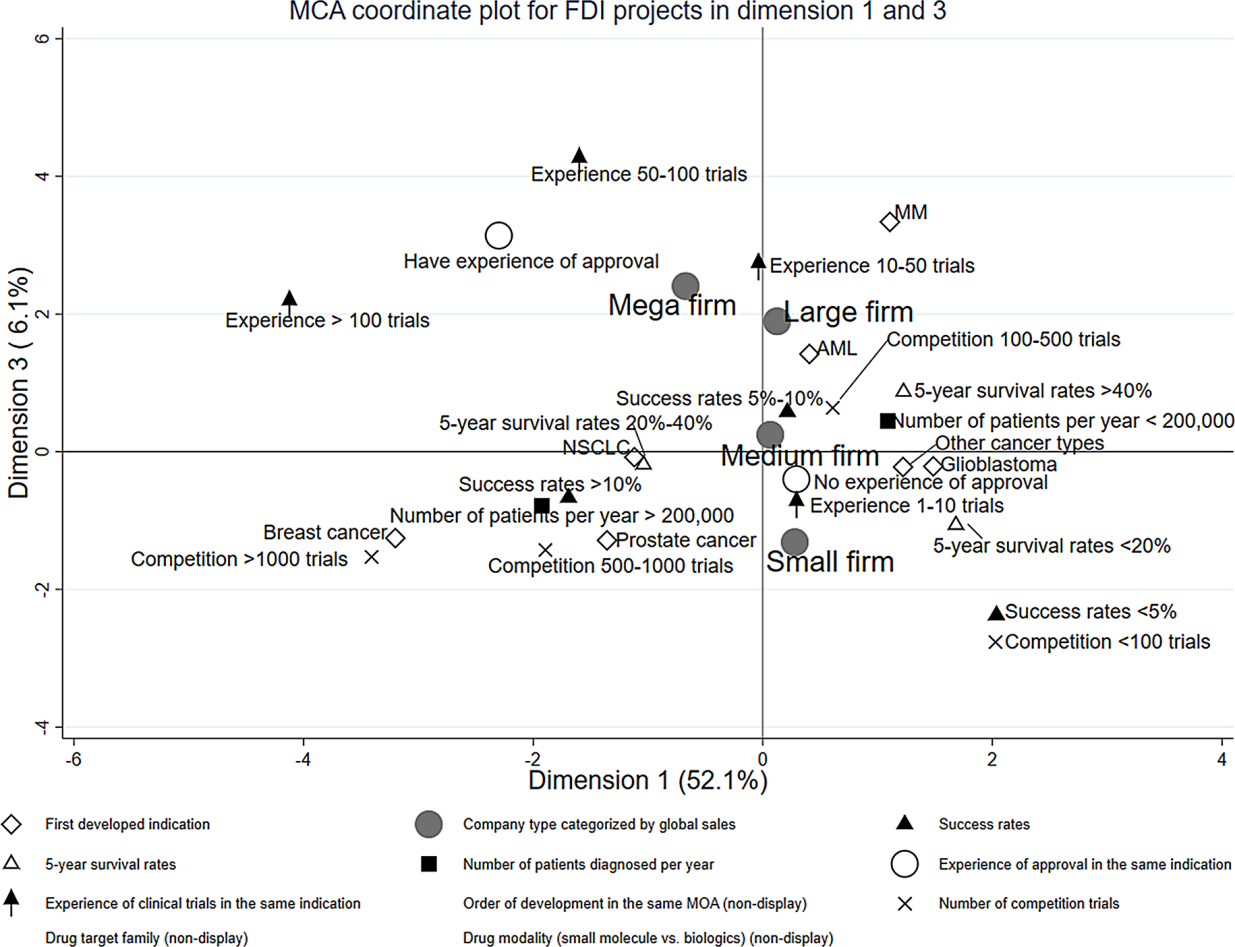
Multiple correspondence analysis coordinate plot for first developed indication (FDI) projects in dimension 1 and 3

**Fig. 5:**
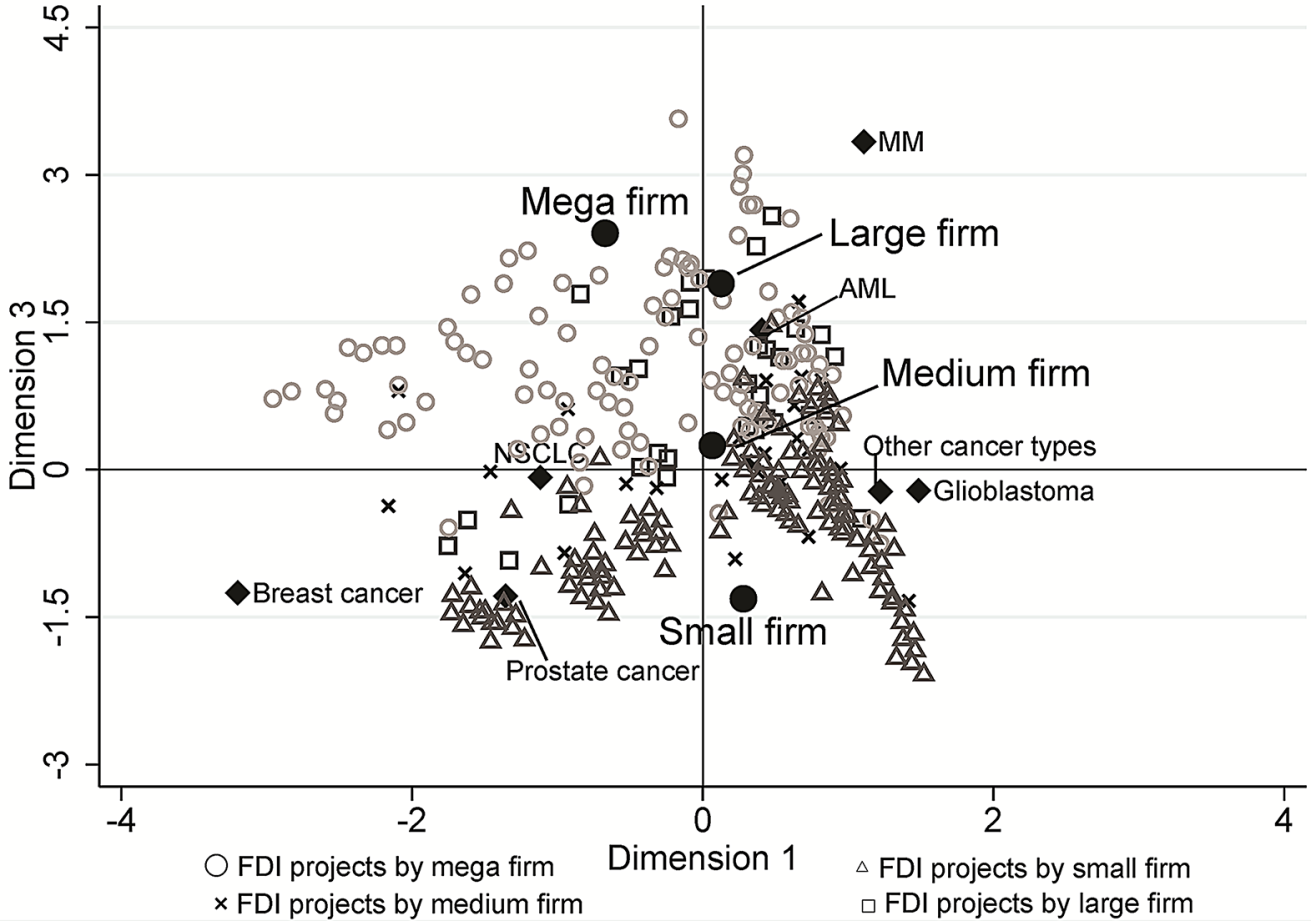
Plots of individual first developed indication (FDI) projects, cancer types and company types in multiple correspondence analysis dimension 1 and 3

We visualized how the individual first development projects were positioned within the distribution of overall projects, applying the multiple correspondence analysis (MCA, Figs. 2, 3, 4 and 5). Three dimensions (i.e., perspectives) were detected that could characterize most efficiently the distribution of the projects, which explains 71.0% of the variation in project characteristics.

The first dimension, contributing most to explaining the total inertia (52.1%), seems to represent ‘the size of expected profits and/or markets’ for the lead indication (horizontal axis in Fig. 2). On the negative side of this axis exist many projects described by the cancer-type characteristics of ‘a large number of patients diagnosed per year, ‘high development competition,’ ‘high probability of development success,’ and ‘medium five-year survival rates.’ First development projects with major cancers such as NSCLC, prostate cancer, and breast cancer are located in the negative region. In contrast, AML, MM, glioblastoma, and other cancer types are located in opposite regions. Information for number of diagnosed patients per year of each cancer type is shown in Table S4. Mega-firm projects are mainly plotted in the negative region. Projects of small firms are relatively more plotted in the opposite (i.e., positive) region, but some are also plotted in the negative region. These results are reflected in the location of the corresponding category centroids on the horizontal axis in Fig. 3.

The second dimension, the explanatory contribution with 12.8%, is interpreted as the axis that refers to ‘novelty for the developer.’ The positive region of the dimension is characterized by a ‘lack of experience in the same indication (e.g., no approved drug for the same indication, little development experience for the same indication)’ (the vertical axis in Fig. 2). Prostate cancer, NSCLC, AML, and glioblastoma were in the positive region, and MM, breast cancer, and other carcinomas were in the negative region. Interestingly, breast cancer is located in the opposite region to NSCLC and prostate cancer. However, these three cancer types have relatively large numbers of patients, indicating that NSCLC and prostate cancers tend to be developed by firms with relatively little experience in developing these cancers. In contrast, breast cancer tends to be developed by firms with relatively extensive experience in developing breast cancer. The category centroids of ‘small firm’ and ‘mega firm’ are plotted on the positive and negative region, respectively (vertical axis in Fig. 3).

Although the contribution in explaining the variability is limited (6.1%), the third dimension likely reflects a ‘tendency to avoid extremes.’ The profiles of ‘moderate (rather than extreme) probability of success,’ ‘moderate (rather than extreme) development competition,’ ‘having an approved drug with the same indication,’ and ‘high five-year survival rates’ appear on the positive side of the vertical axis in Fig. 4. Mega and large firms’ projects are markedly plotted in the positive region, whereas small firm projects are plotted in the negative region (vertical axis in Fig. 5).

Drug novelty (i.e., order of appearance in the same MOA), target molecules, and drug modality did not contribute significantly to the interpretations of the axes.

Plots of FDIs for each cancer type are displayed in different positions on these axes (Figure S1 and S2), solidifying the interpretation of each axis above. Projects for major cancer types, such as NSCLC, prostate, and breast cancer, are plotted in areas where the market is large (the first axis), as expected. The plotting on the second axis reflecting the company’s experience suggests that breast cancer projects are developed by companies with extensive past development experience in the same indication. In contrast, NSCLC and prostate cancer projects are developed by companies with limited past development experience in these same indications. Plots of FDIs for each company type are displayed in Figure S3 and S4. We found no major differences in the distribution of individual projects by firm size on the first and second axes. However, on the third axis, most mega-firm projects are plotted in the ‘avoiding extremes’ area, while smaller firms are plotted in the opposite area.

### Backgrounds Associated with the Choice of Lead Indication

We analyzed how the choice of FDI was related to the drug and firm backgrounds using conditional logit choice models.

Model 1 (Table 2) assumes that all projects may theoretically choose all cancer types as FDI. The regression analysis showed that, regardless of cancer type, companies are more likely to choose indications with past in-house clinical development experience (odds ratio (OR): 2.07–2.19) and marketing experience (OR: 1.49–1.51) in the same indication. Companies avoided indications with higher 5-year survival rates (OR: 0.91). When firm size was divided into four categories (i.e., mega, large, medium, and small), large firms were more likely to choose CLL over NSCLC than mega firms (relative risk ratio (RRR): 4.84–5.59). Medium firms were more likely to choose CLL, colorectal cancer, and hepatocellular cancer over NSCLC than mega firms (RRR: 5.97–12.26). Small firms were less likely to choose CML, small cell lung cancer (SCLC), and melanoma over NSCLC than mega firms (RRR: 0.11–0.48).

**Table 2:** Conditional logit choice model analysis results by Model 1 (with all possible cancer types as choice set)

	Model 1–1 (*N* = 576)			Model 1–2 (*N* = 576)			Model 1–3 (*N* = 576)			Model 1–4 (*N* = 576)		
**Variables**	OR	SE	P-value	OR	SE	P-value	OR	SE	P-value	OR	SE	P-value
Number of competing clinical trials (100)	0.97	0.05	0.514	1.00	0.05	0.522	0.97	0.05	0.568	0.96	0.05	0.504
A company’s experience of approval in the same indication	1.51	0.29	0.031**	1.50	0.29	0.032**	1.50	0.29	0.032**	1.49	0.28	0.036**
A company’s experience of clinical trials in the same indication (100)	2.19	0.62	0.006***	2.16	0.61	0.007***	2.12	0.60	0.008***	2.07	0.59	0.01**
Number of patients diagnosed per year (100,000)				1.14	22.64	0.995						
Observed success rates (%) in the same therapeutic field							1.44	115.89	0.996			
5-year survival rates (%)										0.91	0.02	< 0.01***
**Cancer type (vs. NSCLC)** **only described the ones with statistical difference**												
*Large firm (vs. mega firm)*												
CLL	4.84	4.25	0.073*	5.06	4.46	0.066*	5.27	4.66	0.06*	5.59	4.94	0.052*
*Medium firm (vs. mega firm)*												
CLL	9.69	11.23	0.05*	10.11	11.76	0.047**	10.51	12.25	0.044**	10.97	12.78	0.04**
Colorectal cancer	5.97	6.29	0.09*	6.22	6.58	0.084*	6.63	7.01	0.074*	6.96	7.37	0.067*
Hepatocecullar cancer	9.20	12.81	0.111	10.65	15.15	0.096*	10.88	15.47	0.093*	12.26	17.82	0.085*
*Small firm (vs. mega firm)*												
CML	0.11	0.14	0.07*	0.12	0.14	0.076*	0.13	0.15	0.084*	0.13	0.16	0.093*
SCLC	0.12	0.08	0.002***	0.13	0.09	0.002***	0.13	0.09	0.003**	0.14	0.10	0.004**
Melanoma	0.40	0.21	0.076*	0.42	0.22	0.092*	0.45	0.23	0.116	0.48	0.24	0.149
*First developed product in the MOA (vs. 2nd or later)*												
ALL	6.52	5.99	0.041**	6.60	5.97	0.037**	6.52	5.98	0.041**	6.73	6.19	0.038**
Drug target family is receptor (vs. enzyme)	AML, CLL MM Non-Hodgkin lymphoma, SCLC, glioblastoma, melanoma OR < 1											
Drug modality is biologics therapeutics (vs. small molecule)	MM, Non-hodgkin lymphoma, gastric cancer, ovarian cancer, renal cell cancer OR > 1											
	Log likelihood=-1478.17			Log likelihood=-1478.22			Log likelihood=-1478.26			Log likelihood=-1478.45		

OR, odds ratio; SE, standard error; P-value, probability value; NSCLC, non-small-cell lung cancer; CLL, chronic lymphocytic leukemia; CML, chronic myeloid leukemia; SCLC, small cell lung cancer; MOA, mode of action; ALL, acute lymphocytic leukemia; ALL, acute lymphocytic leukemia; MM, multiple myeloma

* *p* < 0.1. ** *p* < 0.05. ***p < 0.01

First-in-class drugs (i.e., the first drug in each MOA) were more likely to appear in projects for acute lymphoblastic leukemia (ALL) over NSCLC (RRR: 6.52–6.73). Compared with products targeting enzymes, products targeting receptors were less likely to be aimed at hematological cancer (AML, CML, MM, non-Hodgkin lymphoma), SCLC, glioblastoma, and melanoma over NSCLC. Biological drugs tended to appear in projects for MM, non-Hodgkin lymphoma, gastric cancer, ovarian cancer, and renal cell cancer.

Model 1 was based on the assumption that all the cancer types can be chosen. However, cancer types that can be realistically chosen may be limited based on the MOA of the drug and the molecular pathway of the cancer type. Thus, we additionally applied Model 2 (Table S5) which has the constraining assumption that only cancer types chosen by the observed drugs with the same MOA are eligible for choices as FDI. The results were largely consistent with the results from Model 1. Companies tended to select an indication with past clinical development experience (OR: 2.18–2.36) and approval (OR: 1.59–1.60). Cancer types with higher five-year survival rates and higher competition tended to be avoided as FDI (OR: 0.93 and 0.85–0.87, respectively).

The relationships between firm and drug characteristics and the cancer types chosen as FDI found in Model 2 were more or less similar to those found in Model 1.

## Discussion

The results of this study reveal the reality of FDI of the new oncological drugs in the US since 2000. The results of this study have shown an exceptionally high degree of variability in the selection of FDI in terms of the market size of the targeted cancer type, companies’ past development experience, and risks to be accepted for each cancer type. Since this reflects variations in actual decisions of individual developers choosing the most desirable set of a drug and a cancer type when entering the development, the coordinate axes of plots are likely to represent the causes and/or consequences (e.g., a company’s intetion and strategy) of what is considered necessary in the decision.

The main objective of this study was to search for characteristics commonly found in firms’ FDI selection behavior. It is interesting to note that despite the wide variety of projects observed, a common feature of cancer type choice among all projects is the tendency to choose cancer types that have been developed in the past and have a relatively high probability of success.

Our analyses revealed that companies make distinctive first indications based on size (Table 1). For mega-firms, 75 in 102 (65%) projects of solid cancers were focused on major cancer types with high morbidity (e.g., NSCLC, breast cancer, prostate cancer, colorectal cancer, and melanoma). Among hematological cancer projects, 27 in 37 (73%) were for non-Hodgkin lymphoma, CML, and AML. On the other hand, small firms contribute more to projects for rare cancers: 66% of rare solid cancers and 56% rare hematological cancers were developed by small firms.

These differences are also evident in the position of category centroids in the MCA plot. Mega firms tended to enter development with projects targeting larger market-size cancer types with higher mobidity (the horizontal axis in Fig. 2). Smaller firms were more likely to choose cancers other than major cancer types than mega firms. This may underline the justification for interpreting the MCA’s first axis (i.e., market size and/or profit). Given the number of past development projects, it is also not surprising that mega-firms enter development in cancer types in which they have had previous experience compared to smaller firms (the vertical axis in Fig. 2).

It is on the third dimension (the vertical axis of Figs. 3 and 5), which presents the extent of ‘avoiding extremes,’ that the differences in the display of projects between mega and small firms are most clearly evident. The category centroid coordinates of mega and small firm projects are in opposite positions across the origin, and those of large and medium firms fall somewhere between mega and small firms.

On average, the projects of the small firms have an ‘extreme’ profile in terms of development and business risks, while the projects of the mega firms have a ‘moderate’ profile. For example, small firms tend to choose cancer types with very high or very low probability of success, while mega firms tend to choose cancer types in between positions. Mega firms have chosen highly competitive cancer types and moderately competitive areas, while small firms’ choice is skewed toward either highly competitive or less competitive areas. These visualized tendencies are interesting; while many project choices of a small number of mega-firms are similar in targeting relatively moderate profiles, a large number of small firms are highly diverse in their development patterns and attitudes toward risks, each embarking on new projects under very different decision criteria than the mega firms.

The fact that firms of different sizes choose different cancer types is also reflected in the results of the regression analysis (Table 2 and S5). For example, some rare cancer types (e.g., CML, SCLC, and melanoma) were less likely to be chosen by small firms than mega firms. As another example, large and medium firms tended to choose some specific cancer types, including CLL, colorectal, hepatocellular, gastric, and breast cancers. Since only a few firm attributes were available in our analysis, examining the reasons for these imbalances was impossible. However, one possible reason is that firm nationality (or primary target countries/markets) may be confounded in these observations. As observed, projects by Asian companies make up a relatively small percentage of projects by mega and small companies, but about half of projecvts by the medium and large companies. Cancers with a large number of Asian patients (e.g., colorectal cancer, hepatocellular cancer) seem to be chosen more favorably by Asian firms, which could explain some of the observed associations between company size and cancer type.

The results above suggest that firms strategically choose FDI against the background of their respective circumstance; companies with different attributes show different tendency of choices. The next question, then, is whether there are common characteristics that can be seen in the FDI choices of such diverse firms. Our regression analysis revealed three general characteristics common to firms’ choice of cancer types in all first projects. First, firms are more likely to choose cancer types with high development and launch experience; second, cancer types with high five-year survival rates are more likely to be avoided; and third, cancer types with high competition (i.e., many possible entrants) are more likely to be avoided. Although caution must be exercised in interpreting the regression analysis results (‘other conditions being equal’), these characteristics have a certain rationale as the behavior of firms in current new drug development with high uncertainty (i.e., failure risk) and growing costs. They are consistent with the results of previous studies.

Many studies support that experience in the same area of development increases the probability of success for new projects and that companies focus on areas of experience [14–18]. Clinical trials for the development of cancer types with high five-year survival rates take a relatively long time, are generally more difficult, and inflate development costs, so, naturally, they are avoided, other things being equal. The general trend in new drug development in the past decade is avoiding highly competitive cancer types and exploring rare new cancer types that have traditionally been underdeveloped.

Our study focused on analyzing the indication that a company would embark on (i.e., FDI). In our dataset 38.6% of the projects were first approved with different indication from FDI, which means that for quite a few projects FAI is different from FDI. This indicates that many factors must be considered when interpreting FAI, including market competition, priorities in resource allocation, and regulatory processes such as approval review. On the other hand, FDI is more directly linked to the most important objective of obtaining clues about drug efficacy and safety. In particular, the analysis of FDI is considered essential when comparing the behavior of firms with only a few (sometimes only one) development projects with that of large firms, as in this study. The results of this study are useful for future and ongoing consideration of the following objectives. The characteristics and distribution of the current drug development projects provide a predictive picture of how the pharmaceutical market will be structured in the near future. It is of concern to any stakeholder whether this is in the desirable direction regarding efficiency and equity in cancer treatment, and our analysis provides a direct basis for such deliberations. From the perspective of appropriate allocation of social resources, if there is over- or under-entry in certain areas, some intervention (e.g., the provision of incentives) may be necessary to address this. The relationship between the choice of cancer type and firm attributes in the first development project, as revealed by this study, provides a starting point for future support to improve the efficiency of new drug development (e.g., portfolio management) and for real-world policies for effective intervention (if necessary) in the development market as a whole.

This study has several limitations. Firstly, although this study identified some of the background factors in the choice of FDI, it was not aimed to identify the overall mechanism of marketing entry or the process of FDI’s decision. There are multidimensional factors behind firms’ market entry (e.g., firms’ objectives and business conditions, demand for anticancer drugs and medical needs, characteristics of drug candidates to be developed, available technologies). The interpretation of the results of pre-clinical studies should be taken into account in that decision. Studies based on industrial analysis models that consider all of these factors are needed to clarify the mechanisms. Such an investigation is necessary but not possible in the framework of this study, which used only oncological drugs as its sample.

Secondly, consideration of the mechanism linking a new drug candidate’s choice with its chosen indication is also necessary. Whether both choices occur concurrently or whether one precedes the other is an important question that needs to be clarified in itself.

Thirdly, this study focuses on the first step of clinical development, but does not follow the final consequences of the observed projects (i.e., obtaining approvals for inclusion on labels). As seen in the examples where FAI and FDI do not match, uncertainties and LCM strategies not explicitly considered in this analysis may also have an impact on FDI decisions.

## Conclusion

This study analyzed and characterized the first indication companies intend to develop for oncology projects in US after 2000. Companies of different sizes tend to select cancer types with different characteristics when starting clinical development, which reflects the different strategies of the companies behind the selection process. Factors that commonly influence the initiation of development regardless of the size of the company were also observed. It is implicated that companies make development strategies considering their own strengths and market demand as well as competitive risk. The results could provide clues for considering what support measures and incentives are appropriate to balance the efficiency of industrial development and the fulfillment of society’s unmet medical needs.

## Electronic Supplementary Material

Below is the link to the electronic supplementary material.

Supplementary Material 1

Supplementary Material 2

Supplementary Material 3

Supplementary Material 4

Supplementary Material 5

Supplementary Material 6

Supplementary Material 7

Supplementary Material 8

Supplementary Material 9

## Data Availability

No datasets were generated or analysed during the current study.
