# Supplementary figures and images for: Pharmaceutical Company’s Choices of Indication for the First Clinical Projects in Oncological Drug Development in the United States
Source: Ther Innov Regul Sci. 2024 Oct 31;59(1):9–19. doi: 10.1007/s43441-024-00718-2 (PMC11706847; doi:10.1007/s43441-024-00718-2)

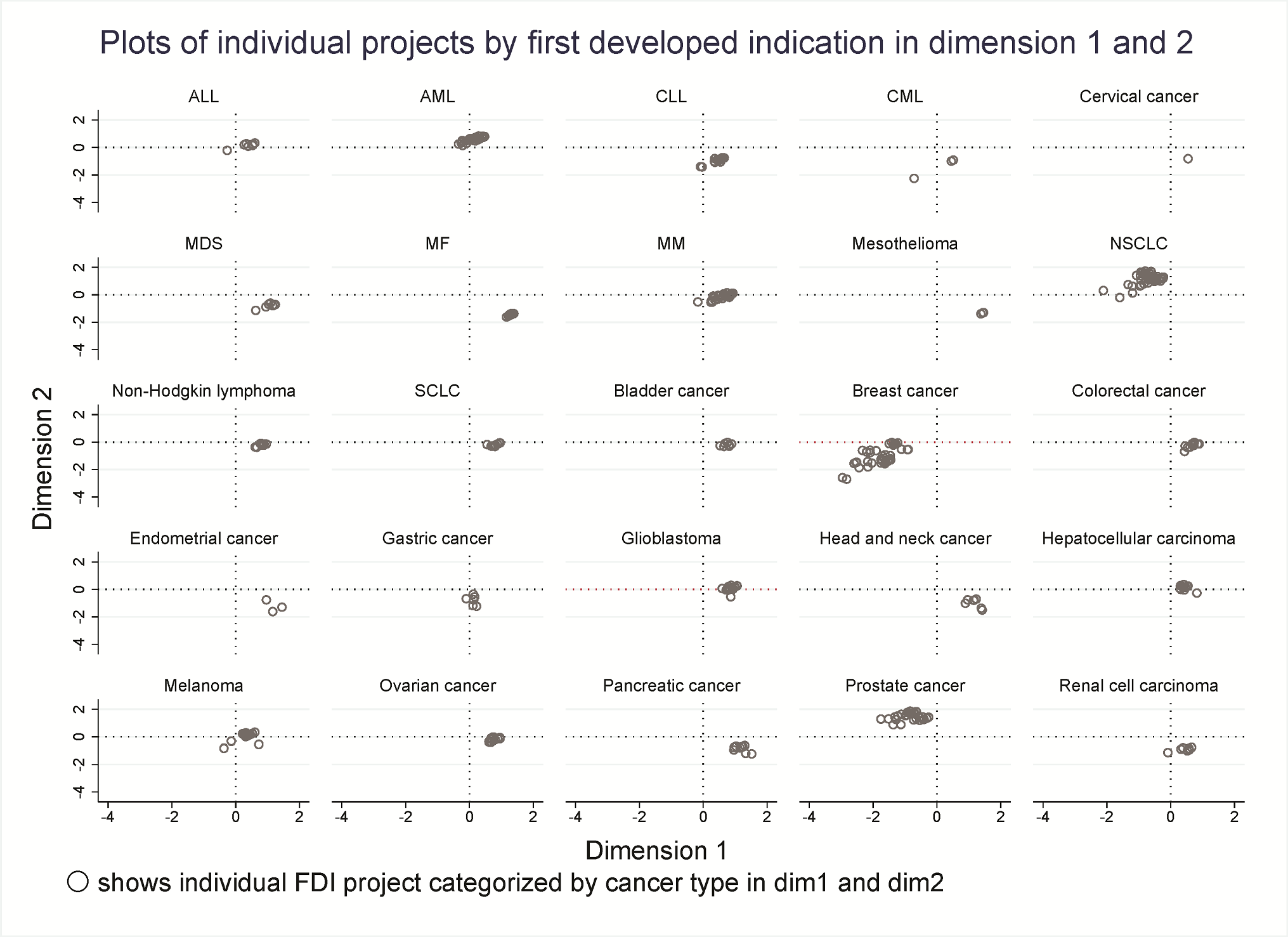

Supplement: Supplementary file 1 — Supplementary Material 1 [file 43441_2024_718_MOESM1_ESM.tif]

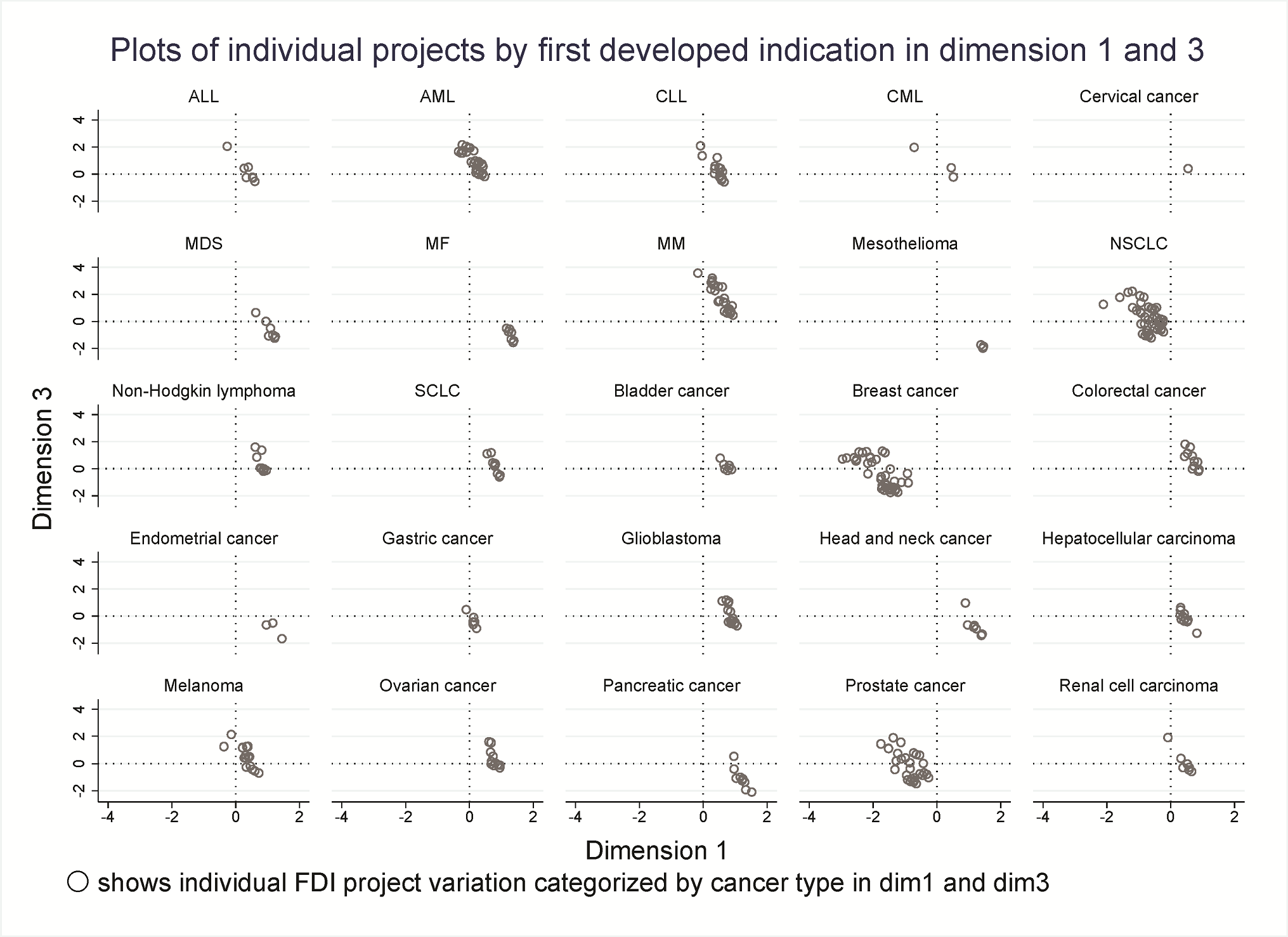

Supplement: Supplementary file 2 — Supplementary Material 2 [file 43441_2024_718_MOESM2_ESM.tif]

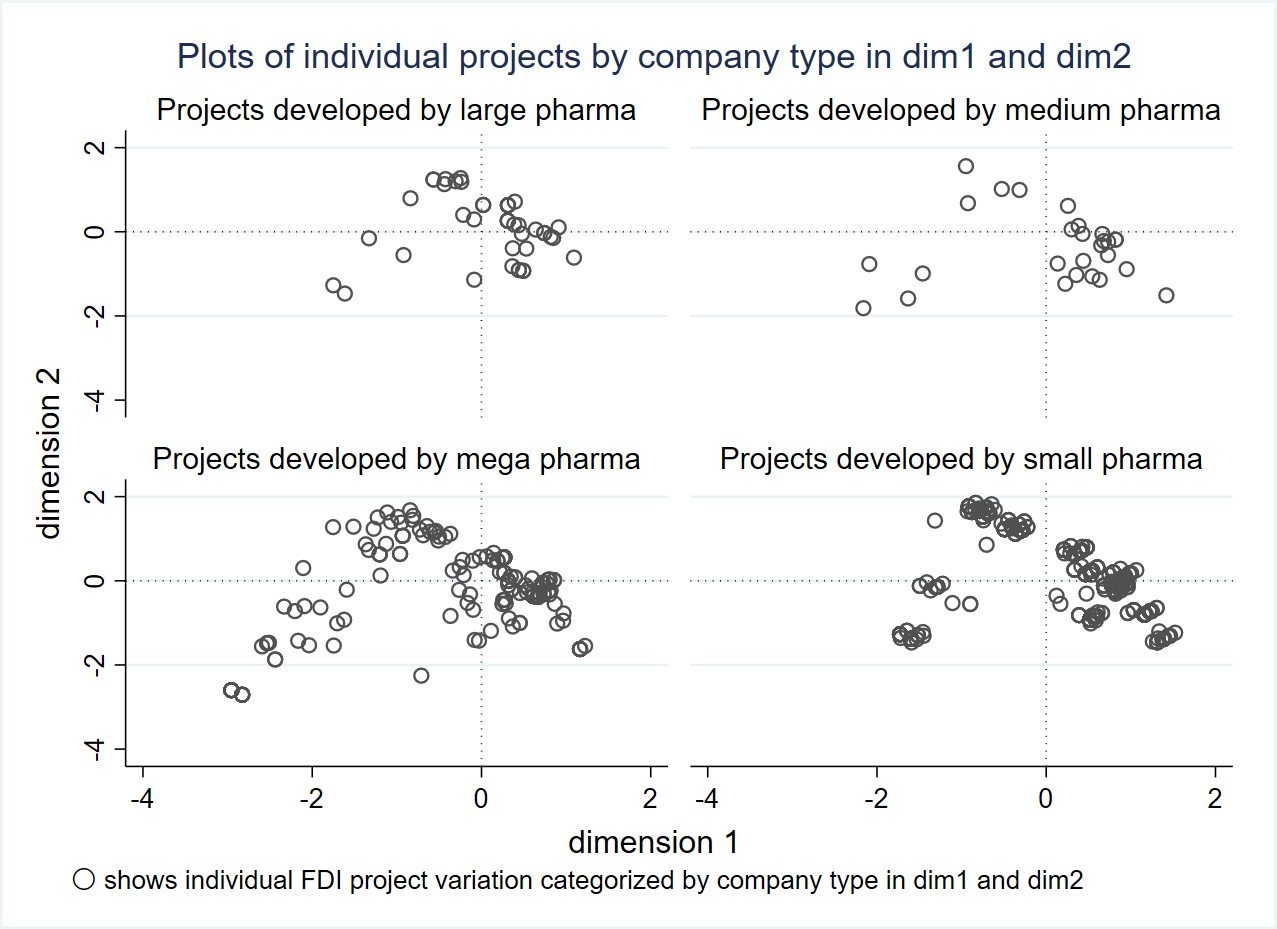

Supplement: Supplementary file 3 — Supplementary Material 3 [file 43441_2024_718_MOESM3_ESM.tif]

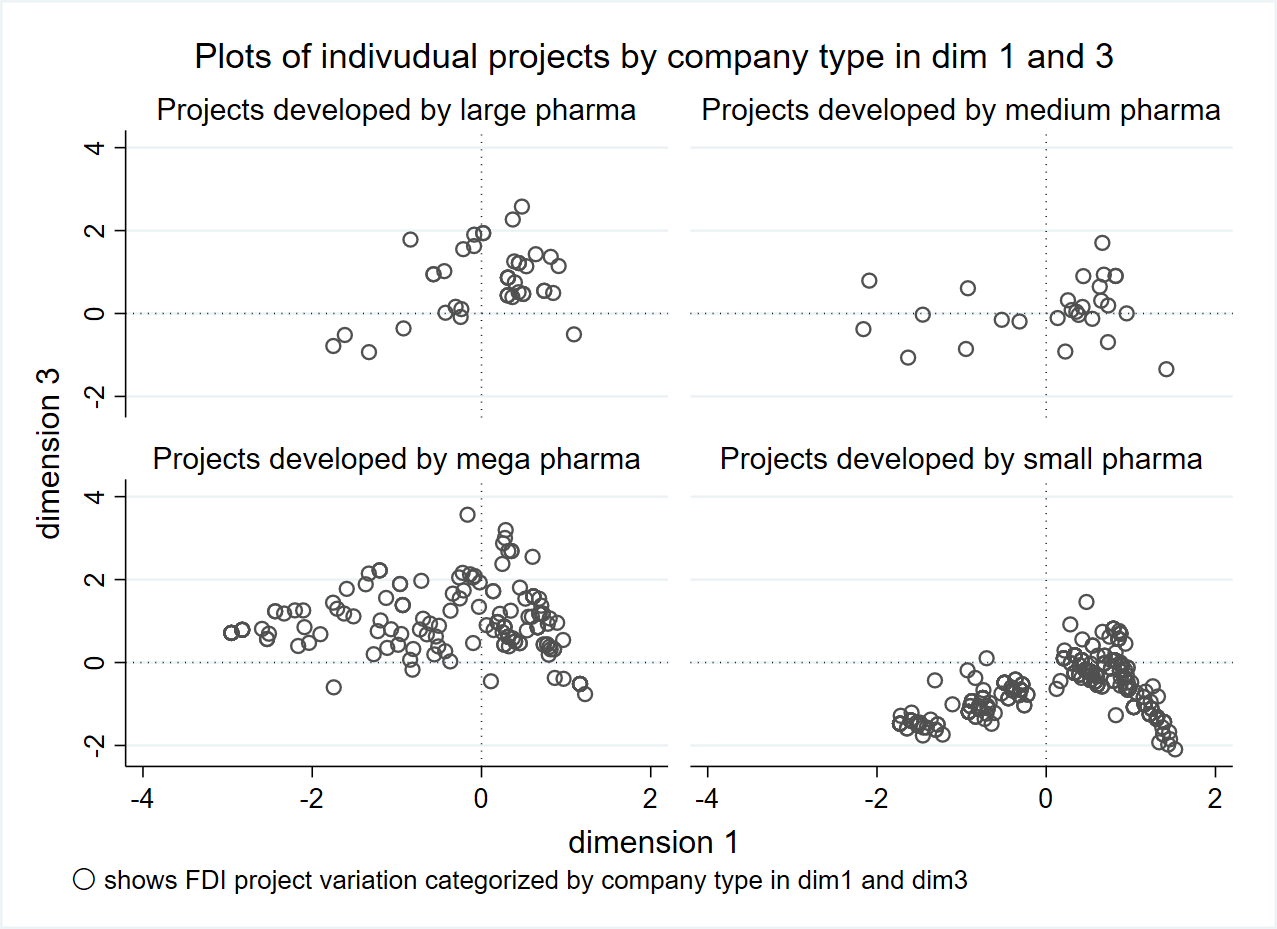

Supplement: Supplementary file 4 — Supplementary Material 4 [file 43441_2024_718_MOESM4_ESM.tif]
