# Supplementary material for: Pharmaceutical Company’s Choices of Indication for the First Clinical Projects in Oncological Drug Development in the United States
Source: Ther Innov Regul Sci. 2024 Oct 31;59(1):9–19. doi: 10.1007/s43441-024-00718-2 (PMC11706847; doi:10.1007/s43441-024-00718-2)
Supplement: Supplementary file 5 — Supplementary Material 5 [file 43441_2024_718_MOESM5_ESM.docx]

Table S1 Variable collecting methods and definition.

| Variables | Definition | Variables in MCA analysis | Variables in regression analysis |
| --- | --- | --- | --- |
| Global sales of a company | Global sales data at the year of development initiation was searched from companies’ annual reports, annual filing of financial statements (i.e., forms 10-K or 20-F) from the database of US Securities and Exchange Commission. If a company has no approved drug, the sales is considered to be 0. If sales data of the year is missing, the nearest year’s data was used in the data set. Considering impact of mergers and acquisitions, if a company has parent company, then the global sales data of the parent company was used. | Mega firms ≧10 billions  Large firms ≧3 billions  Medium firms ≧1 billion  Small firms less than 1 billion | mega firms:1  large firms:2  medium firms:3  small firms:4 |
| Observed success rates in the same therapeutic field | Data was searched from Pharma projects. The definition is Percentage of launched products in overall products in the same therapeutic field（data as of 2022.3）. | ・<5%  ・5%-10%  ・>10% | Observed success rates (%) in pertinent therapeutic fields |
| 5-year survival  rates | Data was searched from Cancer. Net（US）（as of 2021.12） | ・< 20%  ・20%-40%  ・>40%  ・no data | 5-year survival rates (%) |
| Number of patients diagnosed per year | Data was searched from Cancer. Net (US)（as of 2021.12） | ・<50000 person  ・50000-200000 person  ・>200000 person | Number of patients diagnosed per year (per 100,000) |
| A company’s experience of approval in the same indication | Data was searched from FDA approved drugs list. Whether a company had approved drugs or not for the same indication, at the timing of development initiation. | If a company has any approved drug in the pertinent indication, then Yes 1; If a company has no drug in the pertinent indication, then No: 0 | If a company has any approved drug in the pertinent indication, then Yes 1; If a company has no drug in the pertinent indication, then No: 0 |
| Number of competing clinical trials | Data was searched from ClinicalTrials.gov. Number of clinical trials being condudcted in the US for the same indication of “FDI”, at the timing of development initiation for FDI. | ・<100  ・100-500  ・500-1000  ・>1000 | Number of clincal trials (100 trials) |
| A company’s experience of clinical trials in the same indication | Data was searched from ClinicalTrials.gov. Number of clinical trials which were being conducted by the company as sponsor/collaborator, at the timing of development initiation for the drug. | ・1-10  ・10-50  ・50-100  ・>100 | Number of clinical trials (100 trials) |
| Order of development in the same MOA | MOA data was searched from Pharma projects. Line up the start date of clinical trials initiation, and the first developed product was defined as “first in MOA”. Main MOA was counted if there is multiple MOAs for one product.（as of 2021.12） | ・Firs developed product in the MOA: 1  ・Second or later developed product in MOA: 0 | ・Firs developed product in the MOA: 1  ・Second or later developed product in MOA: 0 |
| Drug modality | Drug modality (small molecule vs. biological therapeutic) was searched from database of Pharma projects. | Small molecule vs. biological therapeutic | Biological therapeutic: 1  Small molecule: 0 |
| Drug target family | Drug target family was searched from database of Pharma projects | ・Enzyme  ・Receptor  ・Other drug target families other than enzyme or receptor | ・Enzyme: 1  ・Receptor:2  ・Other drug target families other than enzyme or receptor: 3 |

MCA, multiple correspondence analysis; US, United States; FDI, first developed indication; MOA, mode of action.
