# Supplementary material for: Pharmaceutical Company’s Choices of Indication for the First Clinical Projects in Oncological Drug Development in the United States
Source: Ther Innov Regul Sci. 2024 Oct 31;59(1):9–19. doi: 10.1007/s43441-024-00718-2 (PMC11706847; doi:10.1007/s43441-024-00718-2)
Supplement: Supplementary file 6 — Supplementary Material 6 [file 43441_2024_718_MOESM6_ESM.docx]

Table S2 Herfindahl-Hirschman Index, HHI of cancer types variation for FDI in each MOA.

| MOA | Projects’ number | HHI (%) |
| --- | --- | --- |
| PI3 kinase alpha inhibitor | 27 | 2,277 |
| EGFR kinase inhibitor | 25 | 4,880 |
| Cyclin-dependent kinase inhibitor | 17 | 1,419 |
| DNA synthesis inhibitor | 17 | 1,003 |
| MET tyrosine kinase inhibitor | 15 | 4,666 |
| Histone deacetylase inhibitor | 13 | 1,834 |
| Selective estrogen receptor downregulator | 13 | 10,000 |
| Mitogen-activated protein kinase kinase inhibitor | 12 | 2,361 |
| Poly ADP ribose polymerase inhibitor | 11 | 1,323 |
| Androgen receptor antagonist | 10 | 10,000 |
| Bruton tyrosine kinase inhibitor | 10 | 5,800 |
| Heat shock protein antagonist | 10 | 2,200 |
| VEGFR tyrosine kinase inhibitor | 10 | 1,200 |
| Anaplastic lymphoma kinase inhibitor | 9 | 8,026 |
| Apoptosis stimulant | 9 | 1,848 |
| BET protein inhibitor | 9 | 3,333 |
| FGF receptor tyrosine kinase inhibitor | 9 | 1,728 |
| Immuno-oncology therapy | 8 | 1,563 |
| Janus kinase inhibitor | 8 | 5,938 |
| Protein kinase inhibitor | 8 | 2,500 |
| Tubulin inhibitor | 8 | 2,500 |
| Aurora kinase inhibitor | 7 | 2,653 |
| DNA topoisomerase | 7 | 2,041 |
| Flt-3 antagonist | 7 | 10,000 |
| mTOR kinase inhibitor | 7 | 1,836 |
| Bcl2 inhibitor | 6 | 5,556 |
| B-raf kinase inhibitor | 6 | 5,556 |
| ErbB tyrosine kinase inhibitor | 6 | 5,556 |
| Hedgehog pathway inhibitor | 6 | 1,667 |
| Bcr-Abl inhibitor | 5 | 4,400 |
| Indoleamine 2,7-dioxygenase inhibitor | 5 | 2,800 |
| Isocitrate dehydrogenase inhibitor | 5 | 6,800 |
| Polo-like kinase 1 inhibitor | 5 | 4,400 |
| Proteasome inhibitor | 5 | 10,000 |
| Selective androgen receptor degrader | 5 | 6,800 |
| 17, 20 lyase inhibitor | 4 | 10,000 |
| Bromodomain containing inhibitor | 4 | 3,750 |
| Cereblon E3 ubiquitin ligase stimulant | 4 | 2,500 |
| C-kit inhibitor | 4 | 2,500 |
| K-Ras inhibitor | 4 | 6,250 |
| Lysine (K)-specific demethylase 1A inhibitor | 4 | 5,000 |
| MDM2 inhibitor | 4 | 6,250 |
| Menin-MLL inhibitor | 4 | 10,000 |
| Microtubule modifer | 4 | 6,250 |
| Radiopharmaceutical | 4 | 6,250 |
| Secretase gamma inhibitor | 4 | 3,750 |
| Src inhibitor | 4 | 2,500 |
| Adenosine A2a receptor antagonist | 3 | 3,333 |
| Beta tubulin inhibitor | 3 | 3,333 |
| Checkpoint kinase 1 inhibitor | 3 | 3,333 |
| CXC chemokine receptor antagonist | 3 | 3,333 |
| Estrogen receptor agonist | 3 | 10,000 |
| Fibroblast growth factor receptor 1 antagonist | 3 | 5,556 |
| Focal adhesion kinase inhibitor | 3 | 3,333 |
| IAP antagonist | 3 | 3,333 |
| Kinesin inhibitor | 3 | 3,333 |
| Pim kinase inhibitor | 3 | 3,333 |
| Protein arginine methyltransferase 1 inhibitor | 3 | 3,333 |
| STAT transcription factor 3 inhibitor | 3 | 3,333 |
| Syk tyrosine kinase inhibitor | 3 | 3,333 |
| Toll-like receptor agonist | 3 | 3,333 |
| Transcription factor Nrf2 stimulant | 3 | 3,333 |
| Activin receptor-like kinase 2 inhibitor | 2 | 10,000 |
| Ataxia telangiectasia Rad3 inhibitor | 2 | 5,000 |
| AXL receptor tyrosine kinase | 2 | 5,000 |
| Beta-catenin antagonist | 2 | 5,000 |
| casein kinase inhibitor | 2 | 5,000 |
| CC chemokine receptor 2 antagonist | 2 | 10,000 |
| CD44 antagonist | 2 | 10,000 |
| cell cycle inhibitor | 2 | 5,000 |
| Cyclooxygenase inhibitor | 2 | 5,000 |
| DNA-dependent protein kinase inhibitor | 2 | 5,000 |
| Dopamine D2 receptor antagonist | 2 | 5,000 |
| EGFR antagonist | 2 | 5,000 |
| Endothelin A receptor antagonist | 2 | 10,000 |
| E-selectin antagonist | 2 | 5,000 |
| Glucocorticoid antagonist | 2 | 10,000 |
| Glycogen synthase kinase 3 inhibitor | 2 | 5,000 |
| Hypoxia-inducible factor 2 alpha antagonist | 2 | 5,000 |
| IGF-1 receptor tyrosine kinase inhibitor | 2 | 5,000 |
| Insulin-like growth factor 1 antagonist | 2 | 5,000 |
| p53 stimulant | 2 | 5,000 |
| Platelet-derived growth factor receptor kinase inhibitor | 2 | 5,000 |
| Progesterone receptor antagonist | 2 | 5,000 |
| Prostaglandin EP4 receptor antagonist | 2 | 10,000 |
| Raf kinase inhibitor | 2 | 5,000 |
| RET tyrosine kinase inhibitor | 2 | 10,000 |
| Retinoid receptor agonist | 2 | 5,000 |
| STING agonist | 2 | 5,000 |
| Superoxide dismutase inhibitor | 2 | 5,000 |
| Thymidylate synthase inhibitor | 2 | 5,000 |
| Transforming growth factor beta receptor 1 antagonist | 2 | 5,000 |
| Tyrosine kinase inhibitor (TKI) | 2 | 5,000 |
| Tyrosine phosphatase | 2 | 5,000 |
| VEGF receptor antagonist | 2 | 5,000 |
| Wee-1 tyrosine kinase inhibitor | 2 | 5,000 |
| Adenosine A3 receptor agonist | 1 | 10,000 |
| Alpha2 integrin antagonist | 1 | 10,000 |
| AMPK stimulant | 1 | 10,000 |
| Angiogenesis inhibitor | 1 | 10,000 |
| Apoptosis inhibitor | 1 | 10,000 |
| Arginase inhibitor | 1 | 10,000 |
| ATM kinase inhibitor | 1 | 10,000 |
| ATPase inhibitor | 1 | 10,000 |
| Aryl hydrocarbon receptor antagonist | 1 | 10,000 |
| B-cell maturation antigen antagonist | 1 | 10,000 |
| Benzodiazepine receptor antagonist | 1 | 10,000 |
| caspase 9 | 1 | 10,000 |
| CD166 antagonist | 1 | 10,000 |
| CD22 antagonist | 1 | 10,000 |
| CD71 antagonit | 1 | 10,000 |
| DDX5 inhibitor | 1 | 10,000 |
| Dihydropteroate synthase inhibitor | 1 | 10,000 |
| Dihydrofolate reductase inhibitor | 1 | 10,000 |
| Dihydropyrimidine dehydrogenase inhibitor | 1 | 10,000 |
| Dihydroorotate dehydrogenase inhibitor | 1 | 10,000 |
| DNA synthesis stimulant | 1 | 10,000 |
| Enhancer of zeste homolog 2 inhibitor | 1 | 10,000 |
| Epichaperome inhibitor | 1 | 10,000 |
| Exportin 1 inhibitor | 1 | 10,000 |
| Factor VIIa inhibitor | 1 | 10,000 |
| G2 checkpoint inhibitor | 1 | 10,000 |
| Glutamine antagonist | 1 | 10,000 |
| Glutaminase inhibitor | 1 | 10,000 |
| Glutathione S transferase inhibitor | 1 | 10,000 |
| Growth factor receptor agonist | 1 | 10,000 |
| Heparanase inhibitor | 1 | 10,000 |
| Interleukin-1 receptor associated protein kinase 4 inhibitor | 1 | 10,000 |
| Ion channel antagonist | 1 | 10,000 |
| Kinase inhibitor | 1 | 10,000 |
| Kruppel-like factor 4 stimulant | 1 | 10,000 |
| Leucotriene B4 antagonist | 1 | 10,000 |
| Luteinizing hormone receptor antagonist | 1 | 10,000 |
| MALT1 inhibitor | 1 | 10,000 |
| Map kinase interacting serine/threonine kinase 1 inhibitor | 1 | 10,000 |
| Maternal embryonic leucine zipper kinase inhibitor | 1 | 10,000 |
| Mcl-1 antagonist | 1 | 10,000 |
| Methionine aminopeptidase-2 inhibitor | 1 | 10,000 |
| Myc inhibitor | 1 | 10,000 |
| Na+ K+ transporting ATPase inhibitor | 1 | 10,000 |
| NaP2b antagonist | 1 | 10,000 |
| Nedd 8 activating enzyme inhibitor | 1 | 10,000 |
| Niacinamide phosphoribosyltransferase inhibitor | 1 | 10,000 |
| Notch pathway inhibitor | 1 | 10,000 |
| P glycoprotein inhibitor | 1 | 10,000 |
| P300 inhibitor | 1 | 10,000 |
| p97 inhibitor | 1 | 10,000 |
| Palmitoyl-protein thioesterase inhibitor | 1 | 10,000 |
| Peptidase inhibitor | 1 | 10,000 |
| Peroxisome proliferator-activated receptor alpha antagonist | 1 | 10,000 |
| Porcupine inhibitor | 1 | 10,000 |
| Prostate-specific membrane antigen inhibitor; Tubulin inhibitor | 1 | 10,000 |
| Pyruvate dehydrogenase inhibitor | 1 | 10,000 |
| RAD51 expression inhibitor | 1 | 10,000 |
| Ras inhibitor | 1 | 10,000 |
| Ribonucleoside diphosphate reductase inhibitor | 1 | 10,000 |
| Ribosomal S6 kinase inhibitor | 1 | 10,000 |
| RNA synthesis inhibitor | 1 | 10,000 |
| RORC RAR-related orphan receptor C gamma-t inhibitor | 1 | 10,000 |
| ROS receptor tyrosine kinase inhibitor | 1 | 10,000 |
| Sheddase inhibitor | 1 | 10,000 |
| Signal regulatory protein alpha antagonist | 1 | 10,000 |
| SLAMF7 antagonis | 1 | 10,000 |
| Sphingosine kinase 2 inhibitor | 1 | 10,000 |
| Steroid sulfatase inhibitor | 1 | 10,000 |
| Sulfonamide antibiotic | 1 | 10,000 |
| Superoxide dismutase stimulan | 1 | 10,000 |
| Survivin inhibitor | 1 | 10,000 |
| T cell inhibitor | 1 | 10,000 |
| Taxane | 1 | 10,000 |
| Tumour necrosis factor alpha antagonist | 1 | 10,000 |
| Viral entry inhibitor; | 1 | 10,000 |
| Wnt pathway inhibitor | 1 | 10,000 |
