# Supplementary material for: Pharmaceutical Company’s Choices of Indication for the First Clinical Projects in Oncological Drug Development in the United States
Source: Ther Innov Regul Sci. 2024 Oct 31;59(1):9–19. doi: 10.1007/s43441-024-00718-2 (PMC11706847; doi:10.1007/s43441-024-00718-2)
Supplement: Supplementary file 7 — Supplementary Material 7 [file 43441_2024_718_MOESM7_ESM.docx]

Table S3 Summary of FDI projects categorized by company type and nationality.

| Company type | Number of projects | Projects by Asian companies | Projects by US companies | Projects by Europe companies | Projects by other countries' companies (Canada or Australia) |
| --- | --- | --- | --- | --- | --- |
| Mega firms | 156 | 6 (3.8) | 79 (50.6%) | 71 (45.5%) | None |
| Large firms | 42 | 22 (52.4%) | 10 (23.8%) | 10 (23.8%) | None |
| Medium firms | 27 | 14 (51.9%) | 12 (44.4%) | 1 (3.7%) | None |
| Small firms | 351 | 78 (22.2%) | 235 (67.0%) | 28 (8.0%) | 10 (2.8%) |
