# Supplementary material for: Pharmaceutical Company’s Choices of Indication for the First Clinical Projects in Oncological Drug Development in the United States
Source: Ther Innov Regul Sci. 2024 Oct 31;59(1):9–19. doi: 10.1007/s43441-024-00718-2 (PMC11706847; doi:10.1007/s43441-024-00718-2)
Supplement: Supplementary file 8 — Supplementary Material 8 [file 43441_2024_718_MOESM8_ESM.docx]

Table S4 Characteristics of each cancer type.

| First indication developed | 5-year survival rates | Cancer deaths/year (US) | Diagnosed cases per year (US) |
| --- | --- | --- | --- |
| NSCLC | 25.0% | 112,099 | 200,396 |
| breast cancer (metastatic) | 28.0% | 44,130 | 333,490 |
| prostate cancer (metastatic) | 30.0% | 34,130 | 248,530 |
| glioblastoma | 5.0% | 18,600 | 24,530 |
| melanoma (metastatic) | 27.0% | 7,180 | 106,110 |
| colorectal cancer | 65.0% | 52,980 | 149,500 |
| pancreatic cancer | 10.0% | 48,220 | 60,430 |
| ovarian cancer (metastatic) | 49.0% | 13,770 | 21,400 |
| hepatocellular carcinoma | 20.0% | 30,230 | 42,230 |
| SCLC | 7.0% | 19,782 | 35,364 |
| renal cell carcinoma | 75.0% | 13,780 | 76,080 |
| bladder cancer | 77.0% | 17,200 | 83,730 |
| head and neck cancer | 65.5% | 14,620 | 66,630 |
| gastric cancer | 32.0% | 11,180 | 26,560 |
| endometrial cancer | 81.0% | 12,940 | 66,570 |
| Mesothelioma | 10.0% | 2,500 | 3,000 |
| Cervical cancer | 66.0% | 4,290 | 14,480 |
| AML | 26.0% | 11,400 | 20,240 |
| MM | 54.0% | 12,140 | 34,920 |
| CLL | 86.0% | 4,320 | 21,250 |
| MF | 50.0% | unknown | 4,000 |
| MDS | 14.0% | unknown | 10,000 |
| Non-Hodgkin lymphoma | 73.0% | 20,720 | 81,560 |
| ALL | 38.0% | 1,580 | 5,690 |
| CML | 72.0% | 1,220 | 9,110 |
