# Supplementary material for: Pharmaceutical Company’s Choices of Indication for the First Clinical Projects in Oncological Drug Development in the United States
Source: Ther Innov Regul Sci. 2024 Oct 31;59(1):9–19. doi: 10.1007/s43441-024-00718-2 (PMC11706847; doi:10.1007/s43441-024-00718-2)
Supplement: Supplementary file 9 — Supplementary Material 9 [file 43441_2024_718_MOESM9_ESM.docx]

Table S5 Conditional logit choice model analysis results by Model 2 (with cancer types as choice set limited by MOA).

|  | Model 2-1 (N=437) | | | Model 2-2 (N=437) | | | Model 2-3 (N=437) | | | Model 2-4 (N=437) | | |
| --- | --- | --- | --- | --- | --- | --- | --- | --- | --- | --- | --- | --- |
| **Variables** | OR | SE | P-value | OR | SE | P-value | OR | SE | P-value | OR | SE | P-value |
| Number of competing clinical trials (100) | 0.85 | 0.06 | 0.025** | 0.86 | 0.06 | 0.044* | 0.87 | 0.06 | 0.051* | 0.85 | 0.06 | 0.029** |
| A company’s experience of approval in the same indication | 1.60 | 0.39 | 0.056* | 1.59 | 0.39 | 0.057* | 1.60 | 0.39 | 0.054* | 1.59 | 0.39 | 0.059* |
| A company’s experience of clinical trials in the same indication (100) | 2.36 | 0.92 | 0.027** | 2.19 | 0.00 | 0.043* | 2.25 | 0.87 | 0.036* | 2.18 | 0.84 | 0.045* |
| Number of patients diagnosed per year (100,000) |  |  |  | 1.12 | 40.96 | 0.998 |  |  |  |  |  |  |
| Observed success rates (%) in the same therapeutic field |  |  |  |  |  |  | 1.33 | 251.62 | 0.999 |  |  |  |
| 5-year survival rates (%) |  |  |  |  |  |  |  |  |  | 0.93 | 0.01 | <0.01*** |
| **Cancer type (vs. NSCLC) only described the ones with statistical difference** |  |  |  |  |  |  |  |  |  |  |  |  |
| Large firm (vs. mega firm) |  |  |  |  |  |  |  |  |  |  |  |  |
| CLL | 5.79 | 6.11 | 0.096* | 7.52 | 7.98 | 0.057* | 6.98 | 7.39 | 0.067* | 7.79 | 8.27 | 0.053* |
| Medium firm (vs. mega firm) |  |  |  |  |  |  |  |  |  |  |  |  |
| CLL | 16.44 | 23.65 | 0.052* | 20.26 | 29.30 | 0.037** | 19.05 | 27.52 | 0.041** | 20.44 | 29.54 | 0.037** |
| Colorectal cancer | 5.97 | 6.29 | 0.095* | 9.36 | 12.38 | 0.091* | 9.01 | 11.91 | 0.096* | 9.22 | 12.19 | 0.093* |
| Hepatocecullar cancer | 12.19 | 18.26 | 0.111 | 14.95 | 22.80 | 0.076* | 13.90 | 21.03 | 0.082* | 15.63 | 23.96 | 0.073* |
| Gastric cancer | 18.72 | 29.05 | 0.059* | 21.84 | 33.92 | 0.047** | 20.78 | 32.26 | 0.051* | 22.59 | 35.05 | 0.045** |
| Breast cancer | 4.86 | 4.75 | 0.105 | 5.50 | 5.37 | 0.081* | 5.40 | 5.28 | 0.085* | 5.42 | 5.31 | 0.084* |
| Small firm (vs. mega firm) |  |  |  |  |  |  |  |  |  |  |  |  |
| CML | 0.07 | 0.09 | 0.039* | 0.08 | 0.11 | 0.058* | 0.08 | 0.10 | 0.052* | 0.08 | 0.11 | 0.058* |
| SCLC | 0.05 | 0.04 | <0.001*** | 0.06 | 0.05 | 0.001*** | 0.06 | 0.05 | 0.001** | 0.06 | 0.05 | 0.001** |
| Firs developed product in the MOA  (vs. 2nd or later) |  |  |  |  |  |  |  |  |  |  |  |  |
| ALL | 18.64 | 23.35 | 0.02** | 19.60 | 24.72 | 0.018* | 18.75 | 23.54 | 0.02** | 19.17 | 24.20 | 0.019** |
| CML | 8.97 | 11.44 | 0.086* | 9.29 | 11.84 | 0.08* | 8.95 | 11.42 | 0.086* | 9.45 | 12.05 | 0.078* |
| Drug target family is receptor (vs. enzyme) | AML, breast cancer, melanoma, prostate cancer, Non-hodgkin lymphoma OR <1 | | | | | | | | | | | |
| Drug modality is biologics therapeutics (vs. small molecule) | AML, CLL OR < 1 | | | | | | | | | | | |
|  | Log likelihood= -763.49 | | | Log likelihood= -763.79 | | | Log likelihood= -763.66 | | | Log likelihood= -764.72 | | |

OR, odds ratio; SE, standard error; P-value, probability value; NSCLC, non-small-cell lung cancer; CLL, chronic lymphocytic leukemia; CML, chronic myeloid leukemia; SCLC, small cell lung cancer; MOA, mode of action; ALL, acute lymphocytic leukemia; ALL, acute lymphocytic leukemia; MM, multiple myeloma.

* p<0.1. ** p<0.05. ***p<0.01.
